# Predictors of Cataract Surgery Among US Adults: NHANES 2007–2008

**DOI:** 10.3390/healthcare13060641

**Published:** 2025-03-15

**Authors:** Chisom N. Iwundu, Teija Kohir, Julia E. Heck

**Affiliations:** Department of Rehabilitation and Health Services, University of North Texas, Denton, TX 76203, USA; teijahebshibahkohir@my.unt.edu (T.K.); julia.heck@unt.edu (J.E.H.)

**Keywords:** cataract, occupational, diabetes, blood pressure, aging

## Abstract

Purpose: Cataract, characterized by the clouding of the lens, is the leading cause of blindness and visual impairment worldwide. While cataract surgery is an effective treatment, it carries substantial costs, potential complications, and limited accessibility for those facing financial barriers. Hence, this study aimed to identify the sociodemographic, behavioral, medical, and occupational risk factors of cataract development among adults aged 40 and above. Methods: This cross-sectional study included 2866 participants from the National Health and Nutrition Examination Survey (NHANES) cycle from 2007 to 2008. We utilized a purposeful selection approach to identify the most suitable predictors for cataract surgery. We further used a multivariate logistic regression procedure that accounted for complex sampling design, to assess the main effect of each predictor, entered jointly into the model. Results: Age, blood pressure, and diabetes were identified as predictors of cataract surgery. Each additional year of age increased the odds of undergoing cataract surgery by 15% (OR: 1.15; 95% CI: 1.13–1.16). Participants with high blood pressure had a 38% higher likelihood of cataract surgery (OR: 1.38; 95% CI: 1.11–1.73), while those with diabetes faced a 63% higher likelihood (OR: 1.63; 95% CI: 1.27–2.09). Conclusions: Managing diabetes and blood pressure, especially among older adults, may be crucial in delaying cataract progression.

## 1. Introduction

Cataract, characterized by opacification of the lens, is the leading cause of blindness and visual impairment worldwide [1]. The prevalence of cataract was estimated at 17.2% among US adults over 40 [2]. More than 24 million people had cataract in 2010, a number projected to reach over 50 million by 2050 [3]. This rising prevalence has led to an increase in cataract surgeries. In the U.S, cataract surgery is one of the most commonly performed procedures across all medical specialties, with an estimated 3.7 million cases annually [4]. Although cataract surgery is highly effective in treating cataract, it comes with substantial cost, potential complications, and limited accessibility for individuals facing economic challenges [5]. Therefore, an understanding of the risk factors leading to cataract is crucial for the early identification and management of this condition.

Age is a well-established risk factor for cataract, with age-related cataract being the most common type [6,7,8]. Other identified risk factors include female sex [6] and race/ethnicity (White people have a higher prevalence than Black people) [1,9]. Lower income and educational attainment are associated with reduced access to cataract surgery, reflecting broader healthcare disparities [10,11], while marital status influences the likelihood of surgery, with married individuals more likely to receive cataract surgery, possibly due to better social support [12]. Individuals who are employed or working are also more likely to undergo cataract surgery [13]. Modifiable behavioral factors such as smoking have been consistently related to cataract [14]. In a meta-analysis, smoking was strongly associated with cataract (meta-Odds Ratio (OR): 1.66; 95% confidence interval (CI) = 1.46, 1.89) [15]. Inconsistent evidence has also been shown with alcohol consumption [16]. Medical conditions such as diabetes [6,17], high blood pressure [18], kidney disease [19], and heart disease [20] have been explored, with mixed results. Individuals with diabetes are at high risk of developing cataract at earlier stages of their life when compared to those without diabetes. High glucose can lead to the deposition of sorbitol in the lens that affects naturally occurring proteins, causing it to become less clear and opaque [21]. Cataracts are common among individuals with diabetes, making cataract surgery one of the most frequently performed procedures in this population. The presence of heart disease also often indicates that there are underlying conditions such as high blood pressure and diabetes, which all increase the risk of cataract [22]. Diabetes has been associated with traffic-related air pollution and occupational dust exposure [23,24]. In an exploratory analysis, we include the occupational exposures exhaust fumes, mineral dust, and organic dust as potential correlates. To the best of our knowledge, this is the first study to explore occupational exposures to exhaust fumes, organic dust, and mineral dust in relation to cataract using the NHANES dataset.

Understanding the predictors of cataract, including sociodemographic, behavioral, medical, and occupational factors, can help identify barriers to care and enable the detection of risk factors leading to the earlier development of cataract. While previous studies have examined these predictors individually, few have considered them collectively [6,7,8]. Hence, this study aims to investigate the combined influence of these factors on cataract development among adults aged 40 and above, using data from the 2007–2008 National Health and Nutrition Examination Survey (NHANES).

Declaration of Helsinki: Not applicable.

## 2. Materials and Methods

### 2.1. Study Design and Population

**Figure 1:**
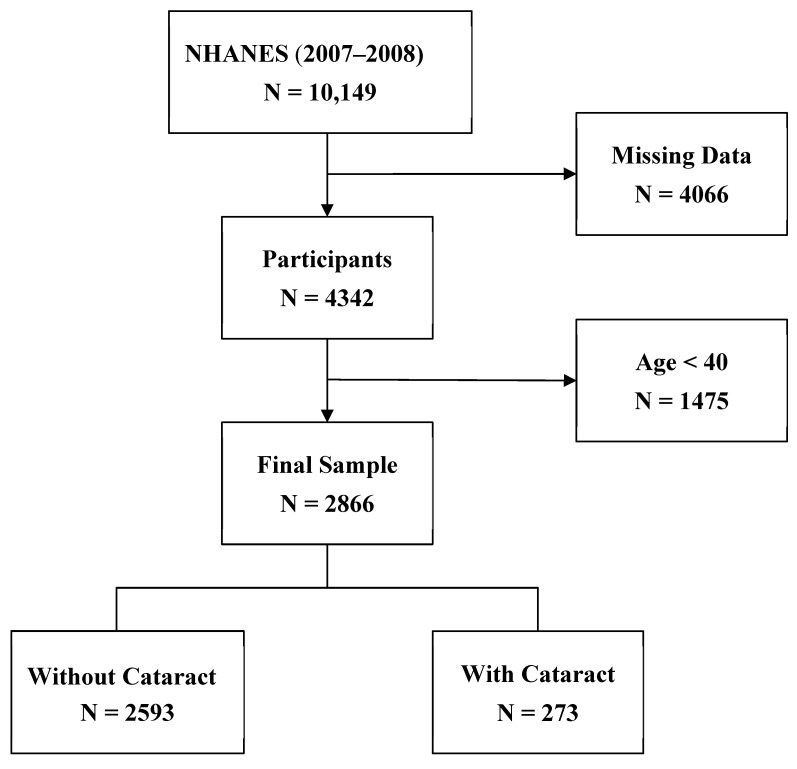
Flowchart of participant selection process.

This study employed a cross-sectional observational design. NHANES is a survey designed to evaluate the health and nutritional status of the U.S population [25]. The survey is conducted by the National Center for Health Statistics at the U.S. Centers for Disease Control and Prevention, and it is unique in combining interviews and physical examinations. The interviews cover demographic, socioeconomic, dietary, and health-related questions, while the physical examinations include medical, dental, and physiological measurements, as well as laboratory tests administered by highly trained medical personnel. We used the 2007–2008 NHANES dataset, focusing on participants older than 39, as cataracts predominantly affect older individuals. The process of participant selection is summarized in Figure 1.

This project did not involve human data or participants; therefore, IRB assessment was not necessary per the policy of the University of North Texas.

### 2.2. Measures

Cataract surgery was used as the measure of cataract occurrence, with the question “Have you ever had eye surgery to treat cataracts (yes or no)?”.

The selection of predictors was guided by the literature [1,6,10,12,15,18,19,20,22,23,26]. We considered age (continuous), gender, race/ethnicity (Mexican American, non-Hispanic White, non-Hispanic Black, Other (includes multiracial), education (less than high school, high school, more than high school, marital status (married/living with a partner, divorced/separated/widowed, never married), income (USD < 35,000, USD 35,000 to USD 75,000, >USD 75,000), employment status at the time of data collection (yes/no), covered by health insurance (yes/no); alcohol, smoking (ever/never); high blood pressure, kidney disease, heart disease (ever/never); exhaust exposure, mineral dust exposure, and organic dust exposure (ever/never). Behavioral factors, including smoking (defined as having smoked at least 100 cigarettes in lifetime) and alcohol consumption (defined as having had at least 12 drinks per year), were dichotomized as (yes or no). Medical conditions were self-reported in response to the question asking whether participants had ever been told they had high blood pressure, diabetes, weak or failing kidneys, or coronary heart disease, respectively.

Descriptive statistics were used to examine the distribution of all variables, stratified by the presence or absence of cataract. A continuous variable, age, was described using mean and standard deviation (SD), while categorical variables were presented as numbers and percentages. To identify the most suitable predictors for cataract surgery, purposeful selection was used. Purposeful selection uses an algorithm to determine variables that should be included for the best model fit. This was carried out using a SAS macro and the process has been previously described [27]. All predictors were added into a model for determination of best fit in the purposeful selection process. At the end, variables deemed to account for the best fit were used for further analyses. We used a multivariate logistic regression procedure that accounted for the complex sampling design to assess the main effect of each predictor, entered jointly into the model. We entered all the predictors jointly so that only the unique variance of each—accounting for the others—would be revealed. SAS v.9.4 was employed for all analyses.

## 3. Results

### 3.1. Participant Characteristics

Table 1 presents the sample characteristics of the participants. The prevalence of cataract surgery in this group was 9% (N = 2866; mean age = 58 ± 10.9). In the overall sample, the gender makeup was 51.1% men and 48.9% women, while the racial distribution of the sample constituted 5.8% Mexican American, 75% Non-Hispanic White, 10.2% Non-Hispanic Black, and 9% Other races. Most of the participants had a college degree or higher (55.5%), were married (70%), earned over $75,000 (35.7%), and were employed/working at the time of data collection (62.8%). Behavioral factors included alcohol consumption and smoking, with the majority of participants being drinkers (72.9%) and smokers (50.3%). Medical conditions explored were relatively low with 40.5% reporting blood pressure, 13.7% diabetes, 2.4% kidney disease, and 4.6% heart disease. The distribution of participants exposed to occupational hazards included 26.5% for exhaust fumes, 33.8% for mineral dust, and 22.4% for organic dust.

Participants with cataract surgery were older (N = 273; mean age 70 ± 7.1), were mostly women (60.9%), and non-Hispanic White (81%), had greater than a high school education (49%), and were married (60%). Unlike results from the total sample distribution, participants with cataract surgery earned an income of less than USD 35,000 (51.3%), and majority were unemployed at the time of data collection (73.9%). Among participants who had cataract surgery, the distribution of the behavioral factors, medical conditions, and occupational exposures, are similar to those of the total sample—high percentages for alcohol consumption and smoking, and lower percentages for medical conditions and occupational exposures.

### 3.2. Predictors of Cataract Surgery

At the end of the purposeful selection process, variables deemed to account for the best fit in predicting cataract surgery were age, blood pressure, and diabetes. Table 2 reports the results of complex sampling multivariate logistic regression model for cataract surgery, with all predictors above entered jointly into the model. Age, blood pressure, and diabetes were identified as predictors of cataract surgery. Each additional year of age increased the odds of undergoing cataract surgery by 15% (OR: 1.15; 95% CI: 1.13–1.16). Participants with high blood pressure had a 38% higher likelihood of cataract surgery (OR: 1.38; 95% CI: 1.11–1.73), while those with diabetes faced a 63% higher likelihood (OR: 1.63; 95% CI: 1.27–2.09).

## 4. Discussion

This study assessed the sociodemographic, behavioral, medical, and occupational predictors of cataract surgery among adults over 39 years, using the 2007–2008 NHANES dataset. The results identified older age, diabetes, and blood pressure as predictors associated with the likelihood of undergoing cataract surgery. While these predictors are consistent with the literature, our study took a different approach, where all assessed predictors were entered jointly into the model as opposed to individually assessing them. In our study, each additional year of age increased the odds of undergoing cataract surgery by 15%. Cataracts are typically a predictable side effect of aging, and our results align with multiple studies that have demonstrated an increased risk of cataracts with advancing age [6,8,28,29,30]. Age-related cataracts may develop due to cellular changes that accumulate over time and ultimately lead to functional impairment [31]. As aging occurs, the lens loses its transparency and ability to focus on near objects. Genetic and environmental factors may also influence these changes, often resulting in cataract. Given that age-related cataracts are typically linked with aging, early detection is crucial to ensure timely treatment to improve overall quality of life [32].

We also identified an association between high blood pressure and the occurrence of cataracts, in concordance with a meta-analysis which estimated an 8% increase in cataract risk with hypertension, estimated from cohort studies [33]. Similarly, our finding is consistent with previous studies that have established diabetes as a risk factor for cataract [6,7,17,34]. Studies such as The Wisconsin Epidemiologic Study of Diabetic Retinopathy [35], The Beaver Eye Dam study [36], and The Blue Mountains Eye Study [37] found associations between diabetes and cataract occurrence. Diabetes is a risk factor for cataract formation, making cataracts the second most common ocular complication associated with the condition [38]. While our study focused on cataract overall, some studies focused on cataract subtypes, finding a risk with cortical cataract [39,40,41]. Cataract was more frequently found in patients with diabetes, and visual improvement was observed in diabetic patients following cataract extraction surgery for advanced cataracts [17]. While we did not measure the duration of diabetes, the longer duration of diabetes has also been attributed to further increasing cataract risk. Therefore, it is important to recommend cataract surgery among diabetics with cataract to allow for visual improvement and assessment and early treatment. Hence, these findings suggest that managing diabetes and blood pressure especially among older adults, may be crucial in delaying cataract progression.

Although our study benefits from a large sample size and a nationally representative population, several limitations should be noted. Due to the cross-sectional design of the study, causality cannot be inferred. Using cataract surgery status as a proxy for cataract may lead to an underestimation of the actual cataract burden. This is because having cataract surgery may reflect individuals with advanced cases, and there might be those presenting with cataract not severe enough to warrant surgery. Although the results of the study are from 2007–2008 data, the findings remain relevant, as the identified risk factors continue to be relevant in current research. Future research using more recent data could further examine how human behaviors have changed over time and their impact on cataract and cataract surgery. To note, our study’s exploration of occupational risk factors is novel, further providing valuable insights. Further, variability in treatment decisions also plays a role, as choices can depend on factors such as access to healthcare, patient preferences, socioeconomic factors, and physician recommendations. Additionally, our study lacks details on cataract type and age of onset. Further, the self-reported nature of the outcome and predictor variables could have resulted in reporting bias; non-differential misclassification would have biased our results to the null. We also selected variables known to be risk factors for cataracts; however, some did not emerge as predictors in our final model (for example, smoking). This may be due to residual confounding, interactions with other covariates, or characteristics specific to our study population. Finally, another limitation is the nature of the predictor variables, which, in some cases, were limited by preordained categories that can also introduce residual confounding (e.g., blood pressure ever, diabetes ever, smoking ever). We, however, utilized purposeful selection to account for the best model fit, and used more than two categories wherever possible (e.g., education, marital status, income). Despite these limitations, the current work extends what is currently known about the predictors of cataract among adults over 39 years old.

## 5. Conclusions

Our study identified age, blood pressure, and diabetes as predictors of cataract surgery in adults over 39, using the 2007–2008 NHANES dataset. While our findings support an association, they do not confirm a direct causal relationship. Thus, longitudinal studies will be valuable to further elucidate the measured associations. Future research should focus on the role of comprehensive management of these conditions in reducing cataract incidence. Public health strategies could also focus on ensuring equitable access to cataract surgery, particularly for underserved populations. Health insurance status and healthcare access are important factors that may influence the likelihood of undergoing cataract surgery. While our study did not directly assess healthcare access, and health insurance was not an identified predictor in our model, these variables remain crucial for understanding the socioeconomic and systemic barriers that may affect cataract surgery utilization. Future research should further explore their impact to provide a more comprehensive view of disparities in surgical access.

The risk factors highlighted in this study underscore the need for preventive care services and targeted interventions to address medical conditions like diabetes and high blood pressure, to improve outcomes for individuals at risk of cataract-related vision impairment.

## Figures and Tables

**Table 1:** Demographic characteristics by cataract surgery, NHANES, 2007–2008, U.S.

Variable	Total	Cataract Surgery	Cataract Surgery
		No	Yes
	N = 2866	(N = 2593)	(N = 273)
	Total N (%)	N (%)	N (%)
*Age* [Mean, SD]	58 [10.9]	56 [10.6]	70 [7.1]
Gender			
Men	1462 (51.1)	1333 (50.4)	129 (39.1)
Women	1404 (48.9)	1260 (49.6)	144 (60.9)
*Race/ethnicity*			
Mexican American	438 (5.8)	414 (6.0)	24 (3.3)
Non-Hispanic White	1434 (75.0)	1269 (74.6)	165 (81.0)
Non-Hispanic Black	601 (10.2)	551 (10.4)	50 (7.9)
Other	393 (9.0)	359 (9.0)	34 (7.8)
*Education*			
Less than high school	853 (18.7)	750 (18.1)	103 (26.3)
High school degree	700 (25.8)	639 (25.9)	61 (24.7)
>High school degree	1313 (55.5)	1204 (56.0)	109 (49.0)
*Marital status*			
Married	1853 (70.5)	1700 (71.3)	153 (60.0)
Divorced/Separated/Widowed	803 (23.1)	694 (22.2)	109 (35.4)
Never Married	210 (6.4)	199 (6.5)	11 (4.6)
*Family Income*			
<USD 35,000	1369 (34.0)	1204 (32.7)	165 (51.3)
≥USD 35,000–USD 74,999	820 (30.3)	746 (30.3)	74 (30.4)
USD 75,000 and over	677 (35.7)	643 (37.0)	34 (18.3)
*Employment at the time of data collection*			
Not working	1367 (37.2)	1149 (34.5)	218 (73.9)
Working	1499 (62.8)	1444 (65.5)	55 (26.1)
*Covered by health insurance*			
No	506 (12.6)	491 (13.4)	15 (2.9)
Yes	2360 (87.4)	2102 (86.6)	258 (97.1)
**Behavioral Predictors**			
*Alcohol Consumption [had at least 12 drinks/year]*			
No	863 (27.1)	764 (26.5)	99 (34.6)
Yes	2003 (72.9)	1829 (73.5)	174 (65.4)
Smoking			
Never	1353 (49.7)	1234 (49.7)	119 (41.0)
Ever	1513 (50.3)	1359 (50.3)	154 (59.0)
**Medical Conditions**			
*High blood pressure ever*			
No	1553 (59.5)	1460 (61.2)	93 (37.0)
Yes	1313 (40.5)	1133 (38.8)	180 (63.0)
Diabetes ever			
* No*	2335 (86.3)	2147 (87.1)	188 (75.0)
* Yes*	531 (13.7)	446 (12.9)	85 (25.0)
*Kidney Condition ever*			
No	2776 (97.6)	2518 (97.8)	258 (94.5)
Yes	90 (2.4)	75 (2.2)	15 (5.5)
*Presence of heart disease ever*			
No	2710 (95.4)	2466 (95.8)	244 (89.1)
Yes	156 (4.6)	127 (4.2)	29 (10.9)
**Occupational Exposures**			
*Exposure to exhaust fumes*			
No	2128 (73.5)	1907 (72.9)	221 (81.5)
Yes	738 (26.5)	686 (27.1)	52 (18.5)
*Mineral Dust*			
No	1913 (66.2)	1709 (65.5)	204 (76.2)
Yes	953 (33.8)	884 (34.5)	69 (23.8)
*Organic Dust*			
No	2246 (77.6)	2017 (77.2)	229 (83.8)
Yes	620 (22.4)	576 (22.8)	44 (16.2)

**Table 2:** Predictors of cataract surgery among adults 40+, NHANES, 2007–2008, U.S. (N = 2866).

Variable	Odds Ratio	95% Confidence Interval
Age	1.15	1.13–1.16
*High blood pressure*		
No	1.00	Referent
Yes	1.38	1.11–1.73
*Diabetes*		
No	1.00	Referent
Yes	1.63	1.27–2.09

## Data Availability

This study utilized data from the National Health and Nutrition Examination Survey (NHANES), a publicly available dataset provided by the Centers for Disease Control and Prevention (CDC). NHANES data can be accessed at https://wwwn.cdc.gov/nchs/nhanes/default.aspx (accessed on 4 March 2025).
